# SUPPORTING DEMENTIA CAREGIVERS IN HEALTHCARE DELIVERY: RECOMMENDATIONS FROM A NATIONAL CONSENSUS CONFERENCE

**DOI:** 10.1093/geroni/igac059.2345

**Published:** 2022-12-20

**Authors:** Catherine Riffin, Joan Griffin, Lauren Bangerter, Lilla Brody, Francesca Falzarano, Karl Pillemer, Sara Czaja, Ronald Adelman

**Affiliations:** Weill Cornell Medical College, New York, New York, United States; Mayo Clinic, Rochester, Minnesota, United States; United Health Group, Edina, Minnesota, United States; Weill Cornell Medicine, New York, New York, United States; Weill Cornell Medicine, Douglaston, New York, United States; Cornell University, Ithaca, New York, United States; Weill Cornell Medicine, New York City, New York, United States; Weill Cornell Medicine, New York City, New York, United States

## Abstract

More than 6 million adults in the United States are affected by Alzheimer’s Disease and related dementias (ADRD), the majority of whom rely on assistance from an unpaid caregiver (family, friends). The goal of the 2021 Conference on Engaging Family and Other Unpaid Caregivers of Persons with Dementia in Healthcare Delivery, funded by the National Institute on Aging, was to establish a policy- and practice-aligned research agenda for enhancing ADRD caregiver engagement and support in healthcare settings. The Conference convened multidisciplinary thought leaders from across the United States to establish a set of actionable recommendations to advance the field. Recommendations centered on five key topics: 1) Identification and assessment of ADRD caregivers, 2) Reimbursement and financing for provider time spent on ADRD caregiver, identification, assessment, and support, 3) ADRD caregiver training and support, 4) Healthcare provider education, and 5) Technology. To support future work in each of the five priority areas, conference participants highlighted the importance of leveraging lessons from implementation science and models of equity and inclusion. Recommendations are intended to inform federal agencies and foundations about high-priority areas and motivate multidisciplinary collaborations to design care delivery systems that effectively engage and support ADRD caregivers.

